# Quantitative Trait Module-Based Genetic Analysis of Alzheimer’s Disease

**DOI:** 10.3390/ijms20235912

**Published:** 2019-11-25

**Authors:** Shaoxun Yuan, Haitao Li, Jianming Xie, Xiao Sun

**Affiliations:** State Key Laboratory of Bioelectronics, School of Biological Science and Medical Engineering, Southeast University, Nanjing 210096, China; 230159460@seu.edu.cn (S.Y.);

**Keywords:** subcortical structure, quantitative trait, module, ADNI, interaction

## Abstract

The pathological features of Alzheimer’s Disease (AD) first appear in the medial temporal lobe and then in other brain structures with the development of the disease. In this work, we investigated the association between genetic loci and subcortical structure volumes of AD on 393 samples in the Alzheimer’s Disease Neuroimaging Initiative (ADNI) cohort. Brain subcortical structures were clustered into modules using Pearson’s correlation coefficient of volumes across all samples. Module volumes were used as quantitative traits to identify not only the main effect loci but also the interactive effect loci for each module. Thirty-five subcortical structures were clustered into five modules, each corresponding to a particular brain structure/area, including the limbic system (module I), the corpus callosum (module II), thalamus–cerebellum–brainstem–pallidum (module III), the basal ganglia neostriatum (module IV), and the ventricular system (module V). Gene Ontology (GO) and Kyoto Encyclopedia of Genes and Genomes (KEGG) enrichment results indicate that the gene annotations of the five modules were distinct, with few overlaps between different modules. We identified several main effect loci and interactive effect loci for each module. All these loci are related to the function of module structures and basic biological processes such as material transport and signal transduction.

## 1. Introduction

Alzheimer’s Disease (AD) is a progressive and irreversible complex neurodegenerative disease which accounts for above 75% of all dementia cases in the world [1]. According to statistics, about 35% of people in the world over 80 years of age suffer from AD [2]. Typical neuropathological features of AD include an increase in the number of senile plaques (SPs) and neurofibrillary tangles (NFTs) [3]. Post-mortem studies have indicated that these neuropathological features originate in the medial temporal lobe limbic system, including the hippocampus and amygdala, and spread to other brain structures as the disease progresses [4]. Therefore, it is necessary to investigate the molecular mechanism of AD from the perspective of multiple brain structures.

AD is a genetically complex neurologic disease; many susceptible genetic loci have been identified by several studies, including those that have used the genetic linkage approach and genome-wide association study (GWAS) [5]. These loci include apolipoprotein E (*APOE*) [6] and other less significant loci such as *BIN1*, *CLU*, *ABCA7*, *CR1*, *PICALM*, *MS4A6A*, *CD33*, *MS4A4E*, and *CD2AP* [7,8]. Single nucleotide polymorphism (SNP) interaction is also of importance in AD research. The effect of a single SNP is small and only a minority of SNPs contribute to the development AD due to “missing heritability” [9]. Recent work on AD has focused on epistasis, which is the interaction between SNPs. Studying these interactions has been reported to have strong potential for revealing the underlying mechanism of AD [10]. One meta-analysis examined the interactions between known AD susceptibility loci and reported a significant interaction between SNP in *APOE* and SNP in *PICALM* [11]. In a whole genome-level interaction analysis, Gusareva et al. found an interaction between rs6455128 (*KHDRBS2*) and rs7989332 (*CRYL1*) [12].

GWAS studies of AD have typically studied qualitative traits. However, studying quantitative traits offers several advantages over qualitative traits, including higher statistical power and smaller required sample size [13]. Quantitative trait-based GWAS is also more objective because the interpretation of results relies on the relationship between identified causative SNPs and hypothetical mechanisms of a particular trait [14]. Quantitative traits can include metabolite concentrations, brain structure volumes, or atrophy rates. For example, *APOE* and 14 other novel genes show significant associations with the cerebrospinal fluid (CSF) amyloid β42 (Aβ42)/total tau (T-tau) concentration ratio [15], and gene *GRIN2B* shows a significant association with temporal lobe volume [16]. Subcortical structures play a critical functional role in basic and higher cognitive ability and are potentially valuable biomarkers of AD [17,18]. Features of subcortical structures, such as their volume, have been frequently used as quantitative traits in AD studies. Most previous quantitative trait-based genetic studies have focused on the implication of genetic loci or loci interactions on subcortical structures. Potkin et al. found five new loci which reached a significant level in the hippocampus atrophy-based GWAS [19]. Moloch et al. performed a gene-set enrichment analysis using statistics from a large-scale genome-wide association study of hippocampal volume [20]. Hibar et al. performed a genome-wide interaction study using two datasets and identified a significant interaction between rs1345203 (*ELF3*) and rs1213205, and this interaction was associated with temporal lobe volume variation [21]. Shashwath et al. tested all SNP interactions within the 212 Kyoto Encyclopedia of Genes and Genomes (KEGG) pathways and identified 125 interactions that may be associated with the right hippocampus atrophy [22].

Multiple subcortical structures work together as modules when performing a specific complex task. A phenotypic modular classification analysis found that structural and functional modules exist in the brain [23]. For example, Chen et al. [24] discovered six modules based on the correlation of cortical thickness; these modules are related to closely overlapping known functional domains, which indicates that functional specialization and integration coexist in the human brain [25]. Thus, analysis of the relationship between subcortical structure modules and genetic factors may reflect the underlying biological mechanism of neurodegenerative disorders such as AD.

The purpose of this study was to investigate the effect of genetic polymorphisms and their interactions on AD-related quantitative traits. Subcortical structure module volumes were used as quantitative traits for the GWAS and SNP interaction analysis. Our study provides a new perspective and goes some way to reveal the relationship between brain structures and genetic factors.

## 2. Results

### 2.1. Sample Demographics

The demographic characteristics of the two groups are shown in Table 1. There was no significant difference in age between the AD group and the normal control group (*p* = 7.12 × 10^−1^). The AD group had significantly fewer years of education than the normal control group (*p* = 3.53 × 10^−6^). The AD group had a significantly lower Mini-Mental State Examination (MMSE) score and a significantly higher Clinical Dementia Rating Sum of Boxes (CDR-SB) score than the normal control group (both, *p* < 2.00 × 10^−16^). The results in Table 1 indicated that AD patients showed marked cognitive dysfunctions compared to normal control subjects.

### 2.2. Module Analysis of Subcortical Structures

**Figure 1:**
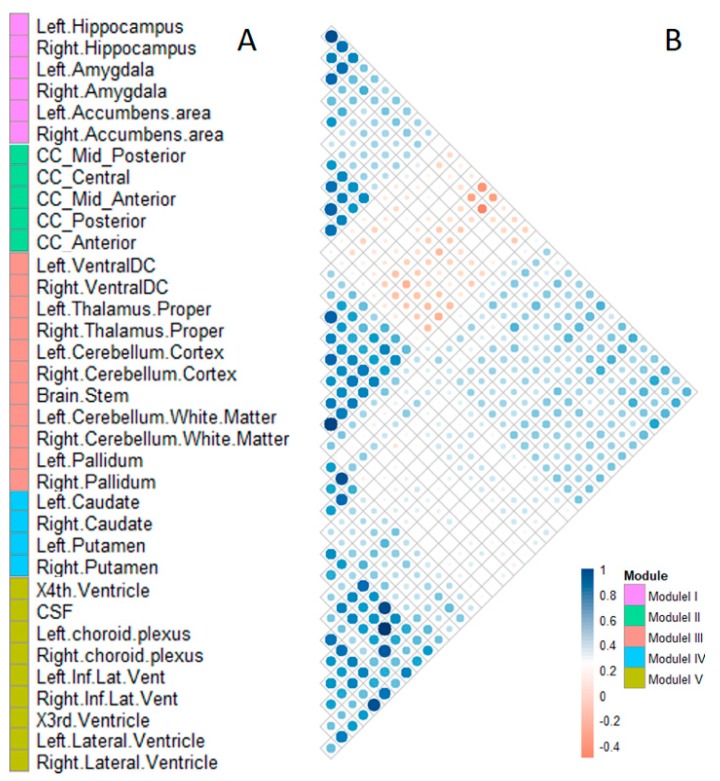
The modular clusters (**A**) and correlation heat map (**B**) of 35 subcortical structures. The color of each cell in the heat map represents the absolute correlation coefficient. Blue indicates a strong correlation and red indicates a weak correlation.

A total of 35 subcortical structures were clustered into five modules according to the Pearson correlation coefficient results. Each module consisted of 4–11 subcortical structures. Modules were labeled from I to V, as shown in Figure 1.

Module I comprised four structures, including the hippocampus, amygdala, and accumbens area, of both hemispheres. Module II included five structures belonging to the corpus callosum. Module III comprised 11 structures, including the ventral-dorsal cord (Ventral DC), thalamus proper, cerebellum cortex, cerebellum white matter, pallidum, and brainstem. Module IV comprised four phenotypes, including the caudate and putamen, in both hemispheres. Module V comprised nine structures, including the choroid plexus, temporal horn of lateral ventricle, lateral ventricle, third ventricle, fourth ventricle, and CSF.

### 2.3. The GO and KEGG Pathway-Enrichment Analysis of Quantitative Trait-Associated SNPs

**Figure 2:**
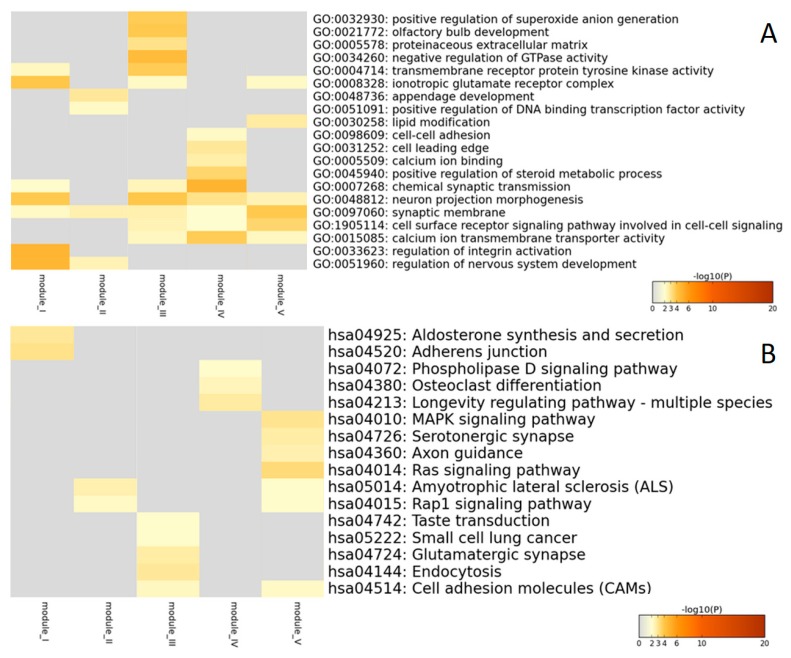
Visualization of Gene Ontology (GO) enrichment results (**A**) and Kyoto Encyclopedia of Genes and Genomes (KEGG) pathway enrichment results (**B**) for modules I–V. The color key from gray to brown represents high to low *p*-values.

To evaluate the biological differences of significant SNPs between modules, we performed Gene Ontology (GO) and KEGG pathway enrichment analyses for the five modules. SNPs with *p*-values smaller than 1.00 × 10^−4^ were identified as significant SNPs in the previous quantitative trait-based GWAS. The Manhattan plots of significant SNPs for each module are shown in Supplementary Figures S1–S5. The numbers of significant SNPs of modules I to V were 367, 310, 270, 534, and 539, respectively, and all these SNPs were mapped to unique genes in the dbSNP database. The numbers of corresponding unique genes were 165, 147, 139, 245, and 241, respectively. The visualization of GO and KEGG enrichment results are shown in Figure 2.

GO and KEGG enrichment results indicated that the gene annotations of the five modules were distinct and with few overlaps between different modules, especially for the KEGG annotation (Figure 2B). The KEGG pathways unique in module I were adheren junctions and aldosterone synthesis and secretion. The KEGG pathways unique in module III were taste transduction, glutamatergic synapse, endocytosis, and small cell lung cancer. The KEGG pathways unique in module IV were phospholipase D signaling pathway, longevity regulating pathway, and the osteoclast differentiation pathway. There were four KEGG pathways unique in module V, including those involved in axon guidance and serotonergic synapses, as well as the Mitogen-activated protein kinase (MAPK) and Ras signaling pathways. Furthermore, module II shared two KEGG pathways with module V, including the amyotrophic lateral sclerosis (ALS) and Rap1 signaling pathway. Module III shared one pathway, cell adhesion molecules (CAMs) pathway, with module V.

### 2.4. Analysis of the Most Significant SNPs and Their Interactions within Each Module

For each module, the top five significant SNPs were identified as the main effect SNPs associated with the quantitative traits of each module (see Supplementary Figures S1–S5). SNP interaction analysis can also provide more information about the pathological mechanisms of AD. Hence, after investigating the main effect SNPs, we further analyzed the top three interactive effect SNPs and their relationships with the quantitative trait of each module. The top three SNPs that have the highest degree with the main effect SNPs were selected as the interactive effect SNPs. The selected SNPs were annotated using the HaploReg program.

#### 2.4.1. The Main Effect and Interactive Effect Loci of Module I

For module I, the main effect SNPs included rs429358, rs6857, rs10414043, rs56131196, and rs2075650 (Table 2). Rs439358 is a nonsynonymous mutation that is located in the coding region of *APOE*. Rs6857 is located in the 3′ Untranslated Region (UTR) region of *NECTIN2*. Rs10414043 and rs56131196 are located in the upstream and downstream regions of *APOC1*, respectively, and are expression Quantitative Trait Loci (eQTL) of *APOC1*. Rs2075650 is located in the intronic region of *TOMM40* and is the eQTL of *TOMM40*. All these main effect SNPs were identified on chromosome 19.

The interactive effect SNPs included rs11121869, rs193067815, and rs76352496, and the degrees of these SNPs were five. Rs11121869 and rs193067815 are located in the intronic region of *TNFRSF8* and *CDH26*, respectively. Rs76352496 is located in the downstream region of *U6*. Mutations of rs11121869 and rs76352496 may cause changes in Transcription Factor (TF) motifs of *TNFRSF8* and *U6*, respectively (Table 3). The top three significant interaction pairs of module I and the other four modules are listed in Supplementary Table S1.

#### 2.4.2. The Main Effect and Interactive Effect Loci of Module II

For module II, the main effect SNPs included rs4646751, rs78018078, rs117336358, rs139169191, and rs146932001 (Table 4). Rs78018078 is located in the intronic region of *CDH18*, while rs139169191 and rs146932001 are located in the downstream region of *CDH18*. Rs4646751 and rs117336358 are located in the intronic regions of *ALDH1L1* and *DENND5A*, respectively (Table 5). Mutation of the main effect SNPs, except rs78018078, may cause changes of TF motifs of their respective genes.

The degrees of the interactive effect SNPs, rs143703214, rs184265794, and rs62184288, were all three. Rs173703214 is located in the intronic region of *FGD4* and may cause changes in TF motifs of *FGD4* (Table 5).

#### 2.4.3. The Main Effect and Interactive Effect Loci of Module III

For module III, the main effect SNPs included rs74545118, rs17757269, rs16924739, rs117508703, and rs7683530 (Table 6). Rs17757269, rs16924739, and rs117508703 are located in the intronic regions of *NRXN3*, *KIAA1217*, and *CYTH1*, respectively. Rs74545118, rs16924739, rs117508703, and rs7683530 are eQTLs of *RAB13*, *KIAA1217*, *CYTH1*, and *RCHY1*, respectively, and mutation of rs17757269 can cause changes of TF motifs of *NRXN3*.

The interactive effect SNPs included rs117497492, rs138637902, and rs142923596. The degrees of these three SNPs were all two. Rs117497492 and rs142923596 are located in the intronic region of *CARHSP1* and *UNC5D*, respectively. Mutations of rs117497492 may cause changes in TF motifs of *CARHSP1* (Table 7).

#### 2.4.4. The Main Effect and Interactive Effect Loci of Module IV

For module IV, the main effect loci included rs189570744, rs72683061, rs111965263, rs137980154, and rs183275415, and only three of these were mapped to genes (Table 8). Rs189570744, rs72683061, and rs137980154 are located in the intronic regions of *CSNK1E*, *ROR1*, and *ARHGEF3*, respectively. Mutation of rs189570744 and rs72683061 may cause changes of TF motifs of *CNSK1E* and *ROR1*, respectively. Rs137980154 affects protein binding of CTCF.

The interactive effect SNPs included rs2800903, rs2592766, and rs149566671. The degrees of these SNPs were all two. Rs2800903 is located in the intronic regions of *ADORA3* and may cause changes of TF motifs of *ADORA3* (Table 9).

#### 2.4.5. The Main Effect and Interactive Effect Loci of Module V

For module V, the main effect SNPs included rs77877165, rs75676610, rs78200466, rs145794994, and rs141634696 (Table 10). Rs75676610 and rs78200466 are both located in the intronic regions of *NGL1*, and rs141634696 is located in the intronic region of *ST3GAL4*. Rs77877165 is located in the downstream region of *NEBL*, while rs145794994 is located in the upstream region of *NNT*. Mutations of all these SNPs may cause changes in TF motifs of their genes.

The interactive effect SNPs included rs2504786, rs9431598, and rs2233857. The degrees of all these SNPs were three. Rs2504786 and rs2233857 are located in the intronic region of *GPR3* and *RFX5*, respectively. Rs2504786 is an eQTL of *GPR3* gene, and mutation of rs2233857 may cause changes of TF motifs of *RFX5* (Table 11).

## 3. Discussion

AD is a progressive, irreversible and genetically complex neurodegenerative disease. Several genetic loci have been identified for AD risk. In this experiment, we performed a quantitative trait module-based genetic study of AD. First, all selected subcortical structures were clustered into five specialized brain area modules according to Pearson’s correlation coefficient. Second, module volumes were used as quantitative traits in the genome-wide association studies for screening the significant SNPs for each module. GO and KEGG pathway enrichment analyses showed few overlaps between different modules, especially for KEGG annotation. Finally, we made detailed analyses of the top five main effect SNPs and three interactive effect SNPs, as well as their relationships with each subcortical structure module and AD.

We clustered 35 selected subcortical structures into five modules. The structures in module I are parts of the limbic system, which is closely related to emotion, memory, and cognition [26], and located in the medial temporal lobe of the cerebrum. The pathological features of the AD first appear in the medial temporal lobe, especially in the hippocampus [27]. The hippocampus–amygdala complex is the most important part of the memory system and plays an important role in human memory function [4]. In addition, the accumbens areas accept large amounts of aggregated fibers from the hippocampus and amygdala and play an important role in several cognitive processes. Module II included five structures belonging to the corpus callosum. The corpus callosum is the largest connective white matter fiber tract in the human brain and contains 200–250 million axonal projections that connect the left and right hemispheres. Diffusion tensor imaging (DTI) findings of patients with AD had a lower fractional anisotropy (FA) value of white matter fibers in the corpus callosum than normal control samples indicate that this structure causes the bilateral spread of AD within the brain [28]. The atrophy of the corpus callosum is a useful biomarker for the diagnosis of AD [29]. Module III comprised 11 structures, including the ventral-dorsal cord (Ventral DC), thalamus proper, cerebellum cortex, cerebellum white matter, pallidum, and brainstem. The pallidum connects the cerebral cortex and thalamus, and the thalamus is the major component of the ventral diencephalons, which plays an important role in consciousness levels. The brainstem, thalamus, and cerebellum are associated with basic life processes, including respiration, heart rate, arousal, movement, balance, and sensation. Module IV comprised four phenotypes, including the caudate and putamen, in both hemispheres. The subcortical structures in this module are involved in independent memory systems belonging to the basal ganglia neostriatum. The neostriatum is an important structure that mainly produces dopamine. There are many dopamine receptors in the dopaminergic system, which are mainly produced in the basal ganglia neostriatum. Module V comprised nine structures, including the choroid plexus, temporal horn of lateral ventricle, lateral ventricle, third ventricle, fourth ventricle, and CSF. These ventricular system structures are related to the storage and transportation of CSF. CSF is an important diagnostic marker of early AD, and the choroid plexus is a plexus of cells that produce CSF in the ventricles of the brain. Several studies have found that biochemical changes in CSF could reflect brain tissue damage, and CSF is an important diagnostic biomarker of AD [30]. According to our results, some subcortical structures have similar patterns of volume changes although they are in different brain areas. For example, caudate, putamen, nucleus accumbens, and pallidum all belong to basal ganglia anatomically, but nucleus accumbens is more similar to hippocampus/amygdala (module I) on volume change patterns, while pallidum is more similar to thalamus and others (module III) on volume change patterns.

We proposed a modular analysis for the human brain subcortical structures in the Alzheimer’s Disease Neuroimaging Initiative (ADNI) cohort. Prior studies have focused on modular analysis on cortical structures, and several modular organizations were discovered [24,31]. To our knowledge, this is the first research on the whole brain subcortical structure module using volumetric measurements. Thirty five subcortical structures were clustered into five modules, each corresponding to a particular brain structure/area. At present, the hippocampus and amygdala in the module I have been widely studied in genetic studies of AD. For example, both volumes of hippocampus and amygdala are associated with *APOE* ε4 in AD patients [32]. Hibar et al. identified four novel loci associated with hippocampal volume [33]. However, there have been few genetic studies on the subcortical structures in modules II, III, IV, and V. In this study, we highlight the need to consider other subcortical structures in AD, including those identified in the other four modules. For this purpose, subcortical structure module volumes were used as quantitative traits for GWAS and interaction analysis.

GO and KEGG pathway enrichment analyses revealed few overlaps between the different modules, especially for the KEGG enrichments, which indicates that the modular clustering was reasonable. KEGG categorizes genes into meaningful biological pathways, and results were more straightforward [34], thus, we only discuss the KEGG results in detail.

Adherens junction proteins are related to maintaining the blood-brain barrier [35] while high plasma concentrations of aldosterone may cause hippocampus dysfunction through the blood-brain barrier [36]. Thus, the two KEGG pathways in module I have both been associated with the blood-brain barrier. Blood-brain barrier disruption has been identified as a key mechanism in the early stages of AD [37]. For module III, degradation of taste and cancer are often considered as complications of AD [38]. For module IV, the activation of phospholipase D (PLD) may be regulated by dopamine receptor D5 [39], which is mainly generated in the neostriatum. For module V, axon guidance might play a role in AD [40] and CSF protein has been reported to participate in the axon guidance signaling pathway [41]. Serotonergic synapses have been associated with memory conditions, and serotonin is abundant in CSF [42]. Kim et al. [43] found that the MAPK signaling pathway is implicated in the development of AD through the regulation of phosphorylation of Amyloid Beta Precursor Protein (APP) and Tau, the main contents in CSF. The Ras and MAPK signaling pathways are activated by the same activity factors such as Grb2 [44]. Module II shares two KEGG pathways, ALS pathway and Rap1 signaling pathway, with module V. ALS is another common neurodegeneration disease as well as AD and Parkinson’s disease (PD) and may involve the same risk pathway as AD and PD [38]. Module III shares one pathway, CAMs pathway, with module V. Multiple studies have found that the CAMs pathway is strongly associated with AD and plays an integral role in the interaction between immune cells and peripheral nerve cells [45].

We selected the top five significant SNPs as the main effect SNPs for each module for further analysis, and the top three interactive effect SNPs were selected from the interaction analysis. In each module, the interactive effect SNPs all interacted with the same subset of the main effect SNPs. The potential pathogenic mechanisms of module-associated genes are listed in Table 12.

For module I, the main effect loci were associated with the volume change of the limbic system. Indeed, *APOE* has been found to be associated with the atrophies of the hippocampus and amygdala [32]. *NECTIN2*, *APOC1*, and *TOMM40* are neighbor genes of *APOE* on chromosome 19 [46,47]. In biology, these loci were involved in the deposition of β-amyloid protein and the abnormal phosphorylation of Tau protein [48]. *TNFRSF8* (Tumor Necrosis Factor Receptor Superfamily Member 8) is associated with neuroinflammation and is a down-regulated gene in the hippocampus from the AD brain but not the normal brain [49]. Neuroinflammation plays a critical role in AD progression and accelerates the development of amyloid-β and tau pathology [50,51]. We speculated that the interactions between *TNFRSF8* and the main effect loci in module I may be involved in the neuroinflammation-induced developments of AD pathological features, which may cause cell death and lead to atrophy of the limbic system in the pathogenesis of AD.

For module II, the main effect loci were associated with the volume change of the corpus callosum and it could be inferred that the abnormal expressions of these loci may cause the atrophy of the corpus callosum. Lines of evidence support our inference. *ALDH1L1* encodes aldehyde dehydrogenase-1 protein, and a lower expression level of *ALDH1L1* may cause white matter damage in AD [52]. *CDH18* encodes calcium-dependent cadherin, and the expression of cadherin plays a key role in the development of neural fiber tracts [53]. *DENND5A* encodes differentially expressed in neoplastic vs normal cells (DENN) domain-containing 5A protein, while its highest level has been found during the development of neuronal development [54]. *FGD4* (FYVE, RhoGEF and PH Domain Containing 4 protein) contains an actin filament-binding domain and is related to cytoskeleton and cell shape. The cytoskeleton is associated with the maintenance of cell shape, and the breakdown of cytoskeletal protein may cause damage to corpus callosum [55]. With these observations, the interactive effect loci *FGD4* may be related to the integrity of corpus callosum, and the underlying regulatory mechanisms need further investigations.

For module III, *RAB13* (Ras-related protein) is a member of small G-proteins. Small G-proteins are regulators of transmembrane transport. *NRXN3* (neurexin 3) is a receptor and cell-adhesion molecule in the central nervous system. *KIAA1217*, also known as sickle tail protein homolog (SKT), is involved in actin binding [56]. *CYTH1* encodes cytohesin-1, which is involved in protein transportation. *RCHY1* encodes E3 ubiquitin ligase and plays an important role in cell proliferation, differentiation, and apoptosis [57]. *CARHSP1* (Calcium Regulated Heat Stable Protein 1) is related to nucleic acid binding and mRNA 3′-UTR binding. *UNC5D* is a netrin receptor and plays a role in cell–cell adhesion and cell guidance. Currently, studies about the genetic factors of AD are more concentrated in the cerebrum, with less attention paid to brainstem, thalamus, and cerebellum. Our result indicated that *RAB13*, *NRXN3*, *KIAA1217*, *CYTH1*, and *RCHY1* were associated with the volume changes of module III, including thalamus, cerebellum, pallidum, and brainstem, and interactive effect loci *CARHSP1* and *UNC5D* may be likely to be associated with basic biological processes.

For module IV, the main effect loci *CSNK1E* and *ROR1* in module IV are associated with the synthesis of dopamine, and the dysfunctions of *CSNK1E* and *ROR1* may cause the atrophy of the basal ganglia neostriatum. Indeed, a decreased level of dopamine in putamen has been proved to be related to the degeneration of dopaminergic neurons, which may lead to the atrophy of the caudate nucleus [58]. *ADORA3* encodes an adenosine A3 receptor protein, a family member of adenosine receptors, which are G-protein-coupled receptors that are involved in a variety of intracellular signaling pathways and physiological functions. Adenosine receptors play a fundamental role in the modulation of dopaminergic neurotransmission [59]. We speculated that the interaction between *ADORA3* and *CSNK1E* has an effect on the reduction of dopaminergic neurons and the atrophy of the basal ganglia neostriatum.

For module V, *NEBL* (nebulette, also called *LASP2*) encodes an actin-binding protein associated with cell attachment, migration, and cellular communication [60]. *NGL1* (netrin G1 protein) is a presynaptic adhesion molecule [61]. *ST3GAL4* (ST3 beta-galactoside alpha-2,3-sialyltransferase-4 protein) is involved in protein glycosylation [62]. *NNT* (nicotinamide nucleotide transhydrogenase) is associated with insulin secretion [63]. *RFX5* encodes a regulatory factor X5, and the homologous gene *RFX6* regulates the production of insulin in the islet [64]. Our results indicated that the interactive effect loci *RFX5* was associated with the secretion of insulin. Higher insulin levels in the brain are correlated with lower rates of whole-brain atrophy in AD [65]. Whole-brain atrophy is related to ventricular enlargement [66]. We speculated that *NNT* and *RFX5* may be involved in the secretion of insulin and the abnormity of these loci may be related to the whole brain atrophy, leading to the enlargements of ventricular system structures in AD. The underly mechanisms of interactions between *RFX5* and *NGL1*, and *ST3GAL4* need further investigations.

## 4. Materials and Methods

Data used in the preparation of this article were obtained from the Alzheimer’s Disease Neuroimaging Initiative (ADNI) database (adni.loni.usc.edu). As such, the investigators within the ADNI contributed to the design and implementation of ADNI and/or provided data but did not participate in analysis or writing of this report. A complete listing of ADNI investigators can be foundat:http://adni.loni.usc.edu/wp-content/uploads/how_to_apply/ADNI_Acknowledgement_List.pdf. The ADNI was launched in 2003 as a public–private partnership, led by Principal Investigator Michael W. Weiner, MD. The primary goal of ADNI has been to test whether serial magnetic resonance imaging (MRI), positron emission tomography (PET), other biological markers, and clinical and neuropsychological assessment can be combined to measure the progression of mild cognitive impairment (MCI) and early Alzheimer’s disease (AD). The authors of this paper were granted approved access to the ADNI data, and the ADNI Data Sharing and Publications Committee (DPC) approved this paper for submission to IJMS (date of approval, October 1, 2019).

### 4.1. Ethics Statement

We used ADNI subject data collected from 50 clinic sites. The ADNI study was conducted according to Good Clinical Practice guidelines, US 21CFR Part 50—Protection of Human Subjects, and Part 56—Institutional Review Boards (IRBs)/Research Ethics Boards (REBs), and pursuant to state and federal Health Insurance Portability and Accountability Act (HIPAA) regulations. Written informed consent was obtained from all participants after they had received a complete description before protocol-specific procedures were carried out based on the 1975 Declaration of Helsinki. IRBs were constituted according to applicable State and Federal requirements for each participating location. The protocols were submitted to appropriate Boards and their written unconditional approval obtained and submitted to Regulatory Affairs at the Alzheimer’s disease Neuroimaging Initiative Coordinating Center (ADNICC) prior to commencement of the study.

### 4.2. Subjects

A total of 393 samples were selected for analysis, including 179 AD samples and 214 normal control (NC) samples. The following data of all ADNI samples were obtained: T1-weighted magnetic resonance imaging (MRI), the Illumina SNP genotyping data, and clinical information of patients including gender, age, years of education, the MMSE score, and the CDR-SB score. The MMSE [67] is a quick and easy measurement for cognitive dysfunction with scores that range from 0 to 30, and the CDR-SB [68] is a clinician-rated staging method that ranges from 0 to 3. Subjects with lower MMSE scores or higher CDR-SB scores indicate greater cognitive dysfunctions.

### 4.3. Quality Control and Imputation of Genotype Data

Quality control processes were applied for samples and SNP data using PLINK software [69]. Samples not meeting any of the following criteria were removed: (1) call rate per sample ≥ 90%; (2) gender check; and (3) identity by descent (IBD) check for related pairs (one sample was excluded from each pair if the IBD was greater than 0.2). SNPs not meeting any of the following criteria were excluded: (1) call rate per SNP ≥ 90%; (2) Hardy-Weinberg Equilibrium test with *p*-value ≤ 5.00 × 10^−7^; and (3) Minor Allele Frequency (MAF) ≥ 10%. Meanwhile, we determined the genotype of rs429358 and rs7412 of *APOE* for each sample using *APOE* ε2/ε3/ε4 information from the ADNI clinical dataset.

Impute2 software [70] was used for missing genotype data imputation. After quality controls and imputation, we performed a genetic data pruned process for SNP data in our experiment. The “indep-pairwise 50 5 0.8” command in PLINK was used to prune the independent SNPs. The purpose of pruning is to remove SNPs that are too much closely-associated with other SNPs, which could screen out a representative SNP from a linkage region and also reduce the time complexity in the following analysis processes.

Finally, 388 samples, including 177 AD patients and 211 NCs, and 2,850,918 SNPs were retained.

### 4.4. Extraction and Clustering of Quantitative Traits

Freesurfer [71] was used for automated segmentation and volume measurement of subcortical structures for all samples from T1-weighted MRI images. Thirty subcortical structures (ROI) (Table 13) and their volumes ($ROI_{raw}^{i}$) were obtained for further analysis. Total intracranial volume (ICV) measurements of each sample were collected from the ADNI database.

A volumetric normalization of subcortical structures was performed due to total head size differences between individuals [72]. Olga [73] proposed a method using a linear regression model to normalize raw structure volume by intracranial volume (ICV), as described below (1). The ICV-normalized structure volume of the *i*th subcortical structure ($ROI_{norm}^{i})$ of a sample was defined as,

(1) $$ROI_{norm}^{i}=ROI_{raw}^{i}-\varepsilon_{i}\left(ICV_{raw}-ICV_{mean}\right)$$

*ICV_raw_* represents the ICV of the sample, and *ICV_mean_* represents the mean ICV across all samples. *ε_i_* is the slope of the regression line between *ROI^i^_raw_* and *ICV_raw_* in the NC group only [74]. *ε_i_* is calculated in the normal control group because the regression slope only represents the normal relationship between brain structure volume and ICV and is not necessarily stable in a pathological patient group.

Pearson correlation coefficients were used to calculate correlations between normalized subcortical structure volumes across all samples. Agglomerative hierarchical clustering was completed using the “complete distance” method to cluster subcortical structures into modules. All subcortical structures were clustered into five modules using cutree parameter ‘cutree_rows = 5′. The correlation matrix heat map and hierarchical clustering were constructed using the pheatmap package in R-project (https://www.r-project.org/). The volume of each module was calculated as the sum of normalized subcortical structure volumes in that module.

### 4.5. Quantitative Trait-Based Genome-Wide Association and Interaction Analyses

SNPs after quality control and imputation and the normalized volumes of each module were used for the quantitative trait-based GWAS and interaction analysis.

GWAS was used to evaluate the association between SNPs and the volume of the module using Plink [69], adjusted for age, gender and education years. Manhattan plots were generated in R using the package qqman. The MatrixEpistasis [75] package in R was used to test the two-way interactions between SNP pairs, adjusted for age, gender and education year.

The top five significant SNPs (the main effect SNPs) in GWAS in each module were selected for further analysis. To define the interactive effect SNPs, first we selected the top five SNPs as main effect SNPs for each module. Then we found other SNPs interacting with main effect SNPs in a genome-wide scale, and interaction pairs with *p*-values smaller than 5.00 × 10^−4^ were kept. Finally, we constructed interaction networks using these interaction pairs, and the top three SNPs that most interacted with main effect SNPs were selected as interactive effect SNPs. HaploReg [76] was used for SNP annotation. HaploReg is a web-based tool for annotating SNPs, including chromosome number, protein binding annotation, and motif change annotation.

The significant SNPs in each module were identified in the dbSNP database (https://www.ncbi.nlm.nih.gov/SNP/). Gene annotations and enrichments visualization for each module were performed using Metascape (http://metascape.org/). In this study, only GO terms and KEGG pathways with a *p*-value smaller than 0.05 were retained.

## 5. Conclusions

With the purpose of investigating the effect of genetic polymorphisms and their interaction on AD-related quantitative traits, we performed a quantitative trait module-based genetic study of AD. To our knowledge, this is the first research on the whole brain subcortical structure module using volumetric measurements. The whole brain subcortical structures were analyzed in our research, giving comprehensive viewpoints of underlying mechanisms of the genetic architecture of complex phenotypes in AD. Subcortical structures were clustered into five anatomical consistent modules, corresponding to the limbic system, corpus callosum, brainstem–thalamus–cerebellum, basal ganglia neostriatum, and CSF system. There were few overlaps of the Gene Ontology and the KEGG pathways among modules, indicating that the modular classification was reasonable.

The main effect and the interactive effect loci for each module were found out. The *APOE* cluster, interacting with *TNFRSF8*, was related to the atrophy of the limbic system through the neuroinflammation. The atrophy of corpus callosum was related to the abnormal expressions of *ALDHL1*, *CDH18*, *DENND5A*, and *FGD4*. Interaction between *ADORA3* and *CSNK1E* had effects on the reduction of dopaminergic neurons and the atrophy of the basal ganglia neostriatum. The abnormities of *NNT* and *RFX5* were related to the whole brain atrophy, leading to the enlargements of ventricular system structures in AD. The limitations of our study are as follows: (1) The sample size of the ADNI dataset used in our study may not provide enough statistical power. Studies with larger sample sizes are required to confirm the role of these loci in AD susceptibility. (2) Our study found several loci that have not been reported to be associated with AD, which needs further investigations.

## Figures and Tables

**Table 1:** Group demographics.

Group	AD	Normal Control	*p*-Value
Number of samples	177	211	-
Gender(Female/Male)	84/93	92/111	-
Age	75.44 ± 7.33	75.67 ± 4.91	7.12 × 10^−1^
Years of Education	14.65 ± 3.17	16.07 ± 2.8	3.53 × 10^−6^
MMSE ^1^ score	23.34 ± 2.09	29.11 ± 0.99	<2.00 × 10^−16^
CDR-SB ^2^ score	4.32 ± 1.67	0.03 ± 0.12	<2.00 × 10^−16^

^1^ MMSE, Mini-Mental State Examination; ^2^ CDR-SB, Clinical Dementia Rating Sum of Boxes.

**Table 2:** The top five main effect SNPs of module I.

SNP ^1^	Chr ^2^	Gene	SNP Annotation	*p*-Value
rs429358	chr19	*APOE*	missense	5.70 × 10^−16^
rs6857	chr19	*NECTIN2*	3′-UTR ^3^	1.40 × 10^−11^
rs10414043	chr19	2.2 kb 5′ of *APOC1*	eQTL ^4^	1.95 × 10^−11^
rs56131196	chr19	239 bp 3′ of *APOC1*	eQTL ^4^	1.25 × 10^−10^
rs2075650	chr19	*TOMM40* (intronic)	eQTL ^4^	5.39 × 10^−9^

^1^ SNP: Single Nucleotide Polymorphism; ^2^ Chr: Chromosome; ^3^ UTR: Untranslated Regions; ^4^ eQTL: expression Quantitative Trait Loci.

**Table 3:** The top three interactive effect SNPs of module I.

SNP ^1^	Chr ^2^	Gene	SNP Annotation	Degree	Interaction
rs11121869	chr1	*TNFRSF8* (intronic)	TF ^3^ motif changed	5	rs429358, rs6857, rs10414043, rs56131196, rs2075650
rs193067815	chr3	*CDH26* (intronic)	-	5	rs429358, rs6857, rs10414043, rs56131196, rs2075650
rs76352496	chr5	54 kb 3′ of *U6*	TF ^3^ motif changed	5	rs429358, rs6857, rs10414043, rs56131196, rs2075650

^1^ SNP: Single Nucleotide Polymorphism; ^2^ Chr: Chromosome; ^3^ TF: Transcription Factor.

**Table 4:** The top five main effect SNPs of module II.

SNP ^1^	Chr ^2^	Gene	SNP Annotation	*p*-Value
rs4646751	chr3	*ALDH1L1* (intronic)	TF ^3^ motif changed	5.74 × 10^−7^
rs78018078	chr5	*CDH18* (intronic)	-	7.15 × 10^−7^
rs117336358	chr11	*DENND5A* (intronic)	TF ^3^ motif changed	1.70 × 10^−6^
rs139169191	chr5	48 kb 3′ of *CDH18*	TF ^3^ motif changed	2.00 × 10^−6^
rs146932001	chr5	93 kb 3′ of *CDH18*	TF ^3^ motif changed	2.00 × 10^−6^

^1^ SNP: Single Nucleotide Polymorphism; ^2^ Chr: Chromosome; ^3^ TF: Transcription Factor.

**Table 5:** The top three interactive effect SNPs of module II.

SNP ^1^	Chr ^2^	Gene	SNP Annotation	Degree	Interaction
rs143703214	chr12	*FGD4* (intronic)	TF ^3^ motif changed	3	rs78018078, rs139169191, rs146932001
rs184265794	chr12	-	TF ^3^ motif changed	3	rs78018078, rs139169191, rs146932001
rs62184288	chr2	-	TF ^3^ motif changed	3	rs78018078, rs139169191, rs146932001

^1^ SNP: Single Nucleotide Polymorphism; ^2^ Chr: Chromosome; ^3^ TF: Transcription Factor.

**Table 6:** The top five main effect SNPs of module III.

SNP ^1^	Chr ^2^	Gene	SNP Annotation	*p*-Value
rs74545118	chr1	*RAB13*	eQTL ^3^	3.28 × 10^−6^
rs17757269	chr14	*NRXN3* (intronic)	TF ^4^ motif changed	4.35 × 10^−6^
rs16924739	chr10	*KIAA1217* (intronic)	eQTL ^3^	4.62 × 10^−6^
rs117508703	chr17	*CYTH1* (intronic)	eQTL ^3^	4.71 × 10^−6^
rs7683530	chr4	*RCHY1*	eQTL ^3^	5.04 × 10^−6^

^1^ SNP: Single Nucleotide Polymorphism; ^2^ Chr: Chromosome; ^3^ eQTL: expression Quantitative Trait Loci; ^4^ TF: Transcription Factor.

**Table 7:** The top three interactive effect SNPs of module III.

SNP ^1^	Chr ^2^	Gene	SNP Annotation	Degree	Interaction
rs117497492	chr16	*CARHSP1* (intronic)	TF ^3^ motif changed	2	rs17757269, rs7683530
rs138637902	chr4	-	TF ^3^ motif changed	2	rs17757269, rs7683530
rs142923596	chr8	*UNC5D* (intronic)	-	2	rs17757269, rs7683530

^1^ SNP: Single Nucleotide Polymorphism; ^2^ Chr: Chromosome; ^3^ TF: Transcription Factor.

**Table 8:** The top five main effect SNPs of module IV.

SNP ^1^	Chr ^2^	Gene	SNP Annotation	*p*-Value
rs189570744	chr22	*CSNK1E* (intronic)	TF ^3^ motif changed	5.55 × 10^−9^
rs72683061	chr1	*ROR1* (intronic)	TF ^3^ motif changed	3.35 × 10^−8^
rs111965263	chr15	-	TF ^3^ motif changed	9.62 × 10^−8^
rs137980154	chr3	*ARHGEF3* (intronic)	Proteins bound of CTCF ^4^	1.06 × 10^−7^
rs183275415	chr8	-	TF ^3^ motif changed	1.83 × 10^−7^

^1^ SNP: Single Nucleotide Polymorphism; ^2^ Chr: Chromosome; ^3^ TF: Transcription Factor; ^4^ CTCF: CCCTC binding factor.

**Table 9:** The top three interactive effect SNPs of module IV.

SNP ^1^	Chr ^2^	Gene	SNP Annotation	Degree	Interaction
rs2800903	chr1	*ADORA3*	TF ^3^ motif changed	2	rs189570744, rs111965263
rs2592766	chr2	-	-	2	rs189570744, rs111965263
rs149566671	chr2	-	-	2	rs189570744, rs111965263

^1^ SNP: Single Nucleotide Polymorphism; ^2^ Chr: Chromosome; ^3^ TF: Transcription Factor.

**Table 10:** The top five main effect SNPs of module V.

SNP ^1^	Chr ^2^	Gene	SNP Annotation	*p*-Value
rs77877165	chr10	114 kb 3′ of *NEBL*	TF ^3^ motif changed	8.03 × 10^−11^
rs75676610	chr11	*NGL1* (intronic)	TF ^3^ motif changed	5.94 × 10^−9^
rs78200466	chr11	*NGL1* (intronic)	TF ^3^ motif changed	5.94 × 10^−9^
rs145794994	chr5	143 kb 5′ *NNT*	TF ^3^ motif changed	6.20 × 10^−9^
rs141634696	chr11	*ST3GAL4* (intronic)	TF ^3^ motif changed	1.08 × 10^−8^

^1^ SNP: Single Nucleotide Polymorphism; ^2^ Chr: Chromosome; ^3^ TF: Transcription Factor.

**Table 11:** The top three interactive effect SNPs of module V.

SNP ^1^	Chr ^2^	Gene	SNP Annotation	Degree	Interaction
rs2504786	chr1	*GPR3* (intronic)	eQTL^4^	3	rs75676610,rs78200466,rs141634696
rs9431598	chr1	-	TF ^3^ motif changed	3	rs75676610,rs78200466,rs141634696
rs2233857	chr1	*RFX5* (intronic)	TF ^3^ motif changed	3	rs75676610,rs78200466,rs141634696

^1^ SNP: Single Nucleotide Polymorphism; ^2^ Chr: Chromosome; ^3^ TF: Transcription Factor; ^4^ eQTL: expression Quantitative Trait Loci.

**Table 12:** Module-associated genes-potential pathogenic mechanisms.

Module	The Main Effect Loci	The Interactive Effect Loci
limbic system	*APOE*, *NECTIN2*, *APOC1*, *TOMM40*: deposition of β-amyloid protein and the abnormal phosphorylation of Tau protein	*TNFRSF8*: neuroinflammation
corpus callosum	*ALDH1L1*, *CDH18*, *DENND5A*: development and integrity of white matter fiber tracts	*FDG4*: integrity of corpus callosum
brainstem, thalamus, and cerebellum	*RAB13*: transmembrane transport; *NRXN3*: cell-adhesion molecule; *KIAA1217*: actin binding; *CYTH1*: protein transportation; *RCHY1*: cell proliferation, differentiation, apoptosis	*CARHSP1*: nucleic acid binding and mRNA 3′-UTR binding *UNC5D*: cell-cell adhesion and cell guidance
basal ganglia neostriatum	*CSNK1E*, *ROR1*: synthesis of dopamine	*ADORA3*: modulation of dopaminergic neurotransmission
ventricular system	*NNT*: secretion of insulin	*RFX5*: secretion of insulin

mRNA: messenger Ribose Nucleic Acid; UTR: Untranslated Regions.

**Table 13:** Freesurfer-defined automated segmentation of subcortical structures.

Phenotype ID	Region of Interest (ROI)
Accumbens-area (L&R)	L&R nucleus accumbens
Amygdala (L&R)	L&R amygdala
Brain-Stem	Brainstem
Caudate (L&R)	L&R caudate nucleus
CC_Anterior	Anterior corpus callosum
CC_Central	Central corpus callosum
CC_Mid_Anterior	Middle anterior corpus callosum
CC_Mid_Posterior	Middle posterior corpus callosum
CC_Posterior	Posterior corpus callosum
CSF	Cerebrospinal fluid
Cerebellum-Cortex (L&R)	L&R cerebellar cortex
Cerebellum-White-Matter (L&R)	L&R white matter of hemisphere of cerebellum
Choroid-plexus (L&R)	Choroid plexus of L&R cerebral hemisphere
Hippocampus (L&R)	L&R hippocampus proper
Inf-Lat-Vent (L&R)	Temporal horn of L&R lateral ventricle
Lateral-Ventricle (L&R)	L&R lateral ventricle
Pallidum (L&R)	L&R globus pallidus
Putamen (L&R)	L&R putamen
Thalamus-Proper (L&R)	L&R thalamus proper
Ventral DC (L&R)	L&R ventral diencephalon
X3rd-Ventricle	Third ventricle
X4th-Ventricle	Fourth ventricle

CC: Corpus Callosum; CSF: Cerebrospinal Fluid; L&R: both left and right hemispheres.

## Supplementary Materials

Supplementary materials can be found at https://www.mdpi.com/1422-0067/20/23/5912/s1.

## Abbreviations

AD	Alzheimer’s Disease
CDR-SB	Clinical Dementia Rating Sum of Boxes
CSF	Cerebrospinal Fluid
GO	Gene Ontology
GWAS	Genome-wide Association Study
KEGG	Kyoto Encyclopedia of Genes and Genomes
ICV	Intracranial volume
MMSE	Mini-Mental State Examination
NC	Normal control
QT	Quantitative trait
ROI	Region of interest
SNP	Single nucleotide polymorphism
