# Supplementary material for: Quantitative Trait Module-Based Genetic Analysis of Alzheimer’s Disease
Source: Int J Mol Sci. 2019 Nov 25;20(23):5912. doi: 10.3390/ijms20235912 (PMC6928939; doi:10.3390/ijms20235912)
Supplement: Supplementary file 1 [file ijms-20-05912-s001.pdf]

Supplementary Table 1. The most significant interaction pairs in each module.

	Main Effect SNPs	Interactive Effect SNPs	Interaction *p*-Value
module I	rs56131196	rs11121869	1.24×10^{-8}
	rs10414043	rs11121869	1.85×10^{-7}
	rs429358	rs11121869	9.19×10^{-7}
module II	rs4646751	rs148020449	1.68×10^{-5}
	rs4646751	rs2561993	1.86×10^{-5}
	rs4646751	rs12875872	2.09×10^{-5}
module III	rs7683530	rs147574874	5.26×10^{-7}
	rs17757269	rs115570732	2.38×10^{-6}
	rs7683530	rs191720284	2.73×10^{-6}
module IV	rs111965263	rs79451801	5.72×10^{-7}
	rs111965263	rs7670325	1.01×10^{-6}
	rs111965263	rs34866539	1.58×10^{-6}
module V	rs75676610	rs11953888	2.58×10^{-7}
	rs78200466	rs11953888	2.58×10^{-7}
	rs75676610	rs139686369	2.71×10^{-7}

SNP: Single Nucleotide Polymorphism.

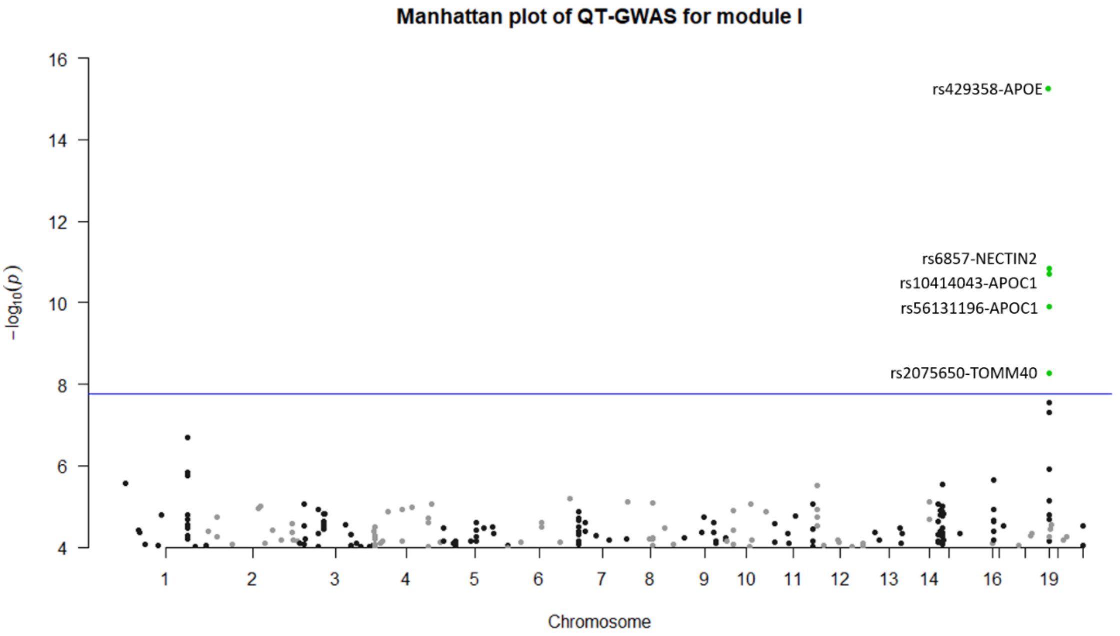

Supplementary Figure 1. Manhattan plot of quantitative trait module-based GWAS for module I. SNPs with a *p*-value smaller than 1.00×10^{-4} are shown in the plot. The blue line represents the Bonferroni-corrected *p*-value (0.05). SNPs marked with a green dot were selected as main effect SNPs.

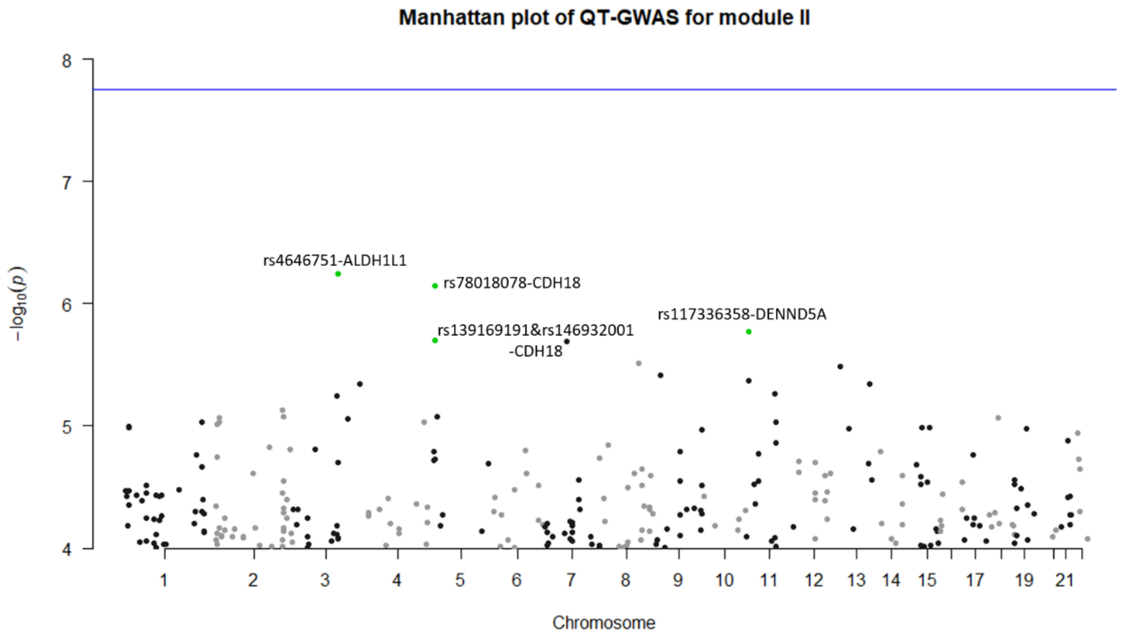

Supplementary Figure 2. Manhattan plot of quantitative trait module-based GWAS for module II. SNPs with a *p*-value smaller than 1.00 × 10^{-4} are shown in the plot. The blue line represents the Bonferroni-corrected *p*-value (0.05). SNPs marked with a green dot were selected as main effect SNPs.

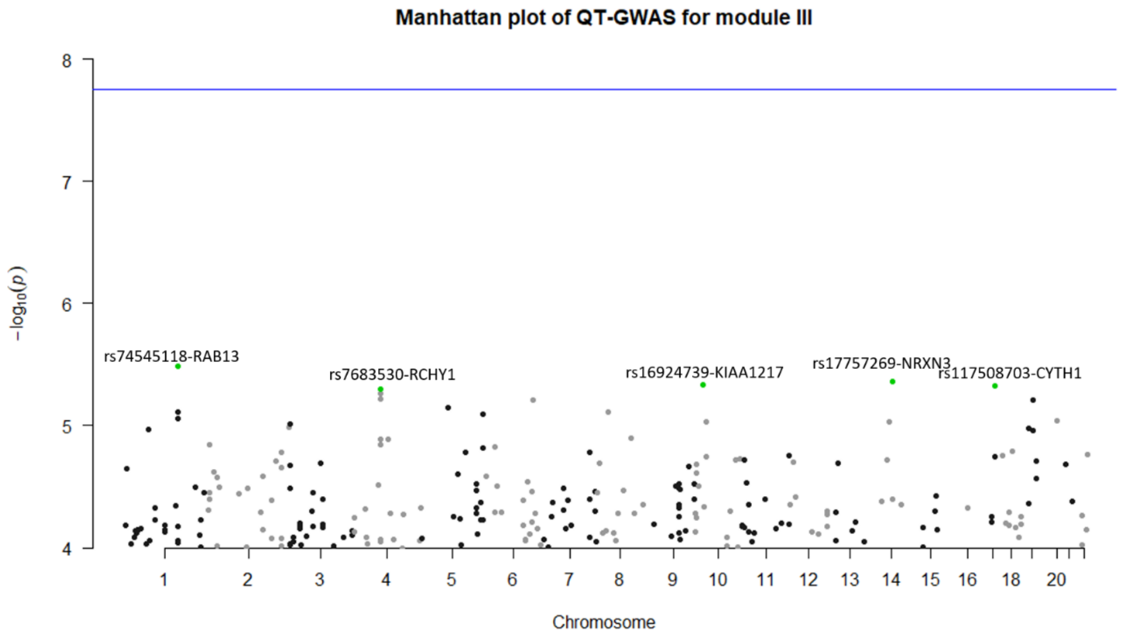

Supplementary Figure 3. Manhattan plot of quantitative trait module-based GWAS for module III. SNPs with a *p*-value smaller than 1.00 × 10^{-4} are shown in the plot. The blue line represents the Bonferroni-corrected *p*-value (0.05). SNPs marked with a green dot were selected as main effect SNPs.

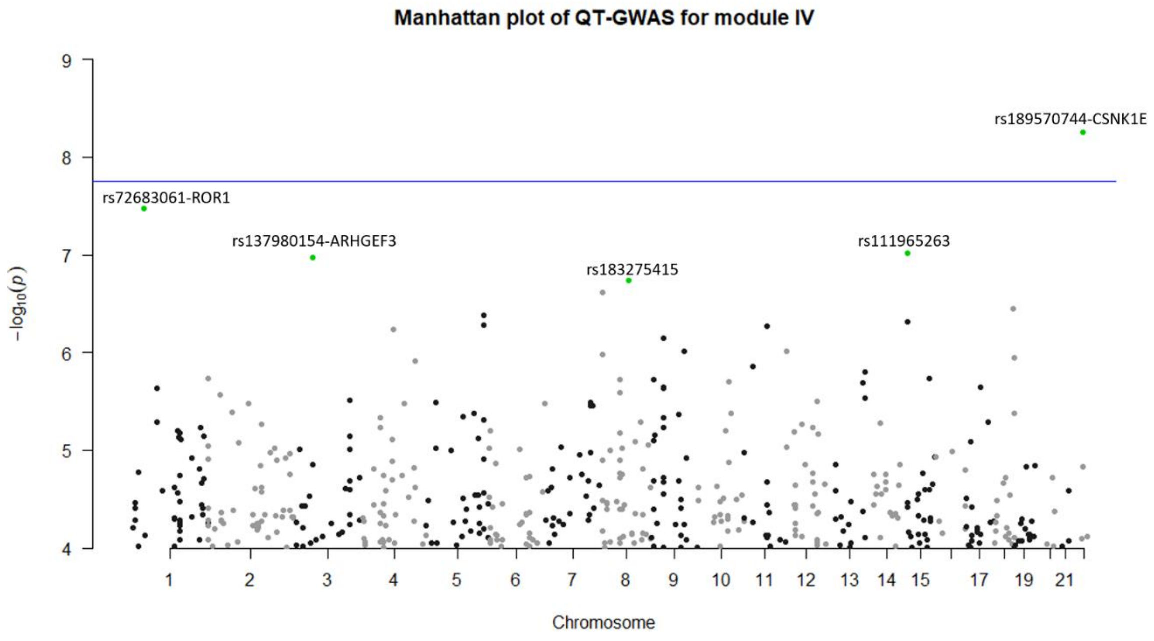

Supplementary Figure 4. Manhattan plot of quantitative trait module-based GWAS for module IV. SNPs with a p-value smaller than 1.00×10^{-4} are shown in the plot. The blue line represents the Bonferroni-corrected p-value (0.05). SNPs marked with a green dot were selected as main effect SNPs.

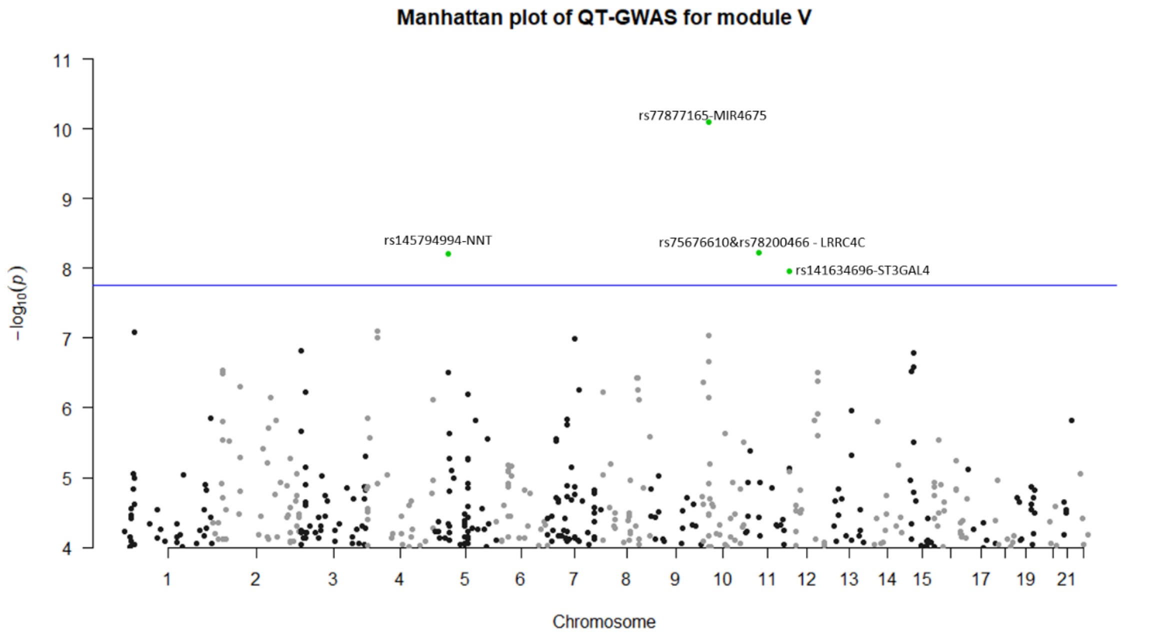

Supplementary Figure 5. Manhattan plot of quantitative trait module-based GWAS for module V. SNPs with a p-value smaller than 1.00×10^{-4} are shown in the plot. The blue line represents the Bonferroni-corrected p-value (0.05). SNPs marked with a green dot were selected as main effect SNPs.
